# Single-nuclei transcriptome analysis of channel catfish spleen provides insight into the immunome of an aquaculture-relevant species

**DOI:** 10.1371/journal.pone.0309397

**Published:** 2024-09-26

**Authors:** Johanna E. Aldersey, Miles D. Lange, Benjamin H. Beck, Jason W. Abernathy

**Affiliations:** 1 Oak Ridge Institute for Science and Education, Agricultural Research Service Research Participation Program, Oak Ridge, TN, United States of America; 2 United States Department of Agriculture, Agricultural Research Service, Aquatic Animal Health Research Unit, Auburn, AL, United States of America; PSTU: Patuakhali Science and Technology University, BANGLADESH

## Abstract

The catfish industry is the largest sector of U.S. aquaculture production. Given its role in food production, the catfish immune response to industry-relevant pathogens has been extensively studied and has provided crucial information on innate and adaptive immune function during disease progression. To further examine the channel catfish immune system, we performed single-cell RNA sequencing on nuclei isolated from whole spleens, a major lymphoid organ in teleost fish. Libraries were prepared using the 10X Genomics Chromium X with the Next GEM Single Cell 3’ reagents and sequenced on an Illumina sequencer. Each demultiplexed sample was aligned to the Coco_2.0 channel catfish reference assembly, filtered, and counted to generate feature-barcode matrices. From whole spleen samples, outputs were analyzed both individually and as an integrated dataset. The three splenic transcriptome libraries generated an average of 278,717,872 reads from a mean 8,157 cells. The integrated data included 19,613 cells, counts for 20,121 genes, with a median 665 genes/cell. Cluster analysis of all cells identified 17 clusters which were classified as erythroid, hematopoietic stem cells, B cells, T cells, myeloid cells, and endothelial cells. Subcluster analysis was carried out on the immune cell populations. Here, distinct subclusters such as immature B cells, mature B cells, plasma cells, γδ T cells, dendritic cells, and macrophages were further identified. Differential gene expression analyses allowed for the identification of the most highly expressed genes for each cluster and subcluster. This dataset is a rich cellular gene expression resource for investigation of the channel catfish and teleost splenic immunome.

## Introduction

The immune system provides protection from invading pathogens and is divided into the innate immune response and the adaptive immune response. The innate immune system is a rapid, generalized response to pathogens, whereas the adaptive immune response is a slower but targeted defense. Additionally, there are immune system agents with both innate and adaptive functions. Teleost fish are an important comparative model for the immune system given that they are lower order vertebrates with both elements of innate and adaptive immune systems [1]. The innate immune system of teleost consists of monocytes/macrophages, granulocytes, dendritic cells (DCs), natural killer (NK) cells and innate lymphoid cells (ILC) [2].

The major lymphoid organs in teleost are the thymus, head kidney and spleen [3]. Like in mammals, the thymus generates and differentiates T cells [3]. The head kidney is the fish equivalent to bone marrow in mammals [3]. The spleen provides an environment for lymphocytes, B and T cells, to exist and proliferate [4]. By maintaining these cells, the spleen also facilitates contact between the lymphocytes and antigens/antigen presenting cells to mount an immune response [4]. Furthermore, the spleen acts as a filter to clear circulating antigens and pathogens from the blood [4] and maintains erythrocyte homeostasis.

Zebrafish (*Danio rerio*) is the most studied teleost immune model. Besides zebrafish, the channel catfish (*Ictalurus punctatus*) has also served as an immunological model for the teleost innate and adaptive immune system [5]. Though compared to mammals, in particular human and mice, complex interactions of the innate and adaptive immunity are not well understood. It is evident that the main mechanisms of the immune system overlap between teleost and mammals [5,6]. However, even within teleost, there are unique attributes. For example, catfish only express IgD and IgM, while IgZ/T isotypes have been detected in zebrafish, salmonids, and other teleost fish [7–10].

The production of channel and hybrid catfish are of great commercial interest as they represent the largest sector of the United States (U.S.) aquaculture industry, with sales generating $437 million in 2023 [11]. Significant losses in production can be attributed to pathogenic diseases, and epidemiological reports show that catfish production is most affected by bacterial diseases such as motile *Aeromonas* septicemia caused by *Aeromonas hydrophila*, columnaris disease caused by *Flavobacterium covae* and enteric septicemia caused by *Edwardsiella ictaluri* [12]. A basic understanding of the catfish adaptive immune system will facilitate investigation into the progression of aquaculture-relevant diseases. Furthermore, the single-nuclei data produced from this study will be used to provide a baseline for studying the effects of active infection and disease interventions such as vaccines.

Studying the immune cell types in catfish and their gene expression profiles has been limited to bulk RNA sequencing (RNAseq) [13–15]. Bulk RNAseq extracts transcriptomic information from a tissue or cell population, and thus the data represents the average gene expression of the cells [16]. Therefore, for samples with heterogenous cell populations, transcriptomic information is mixed, and signals from rare cell types are obscured or not detected [16]. However, the additional characterization of immune cells in the heterogenous lymphoid tissues is now possible with single-cell/single-nuclei RNAseq (scRNAseq/snRNAseq). In sc- and snRNAseq, the transcriptomic analysis occurs at the individual cell level, allowing cell types and states to be studied, and between cell type differences to be characterized [16]. Such technology has already been applied to study zebrafish and Atlantic salmon (*Salmo salar*) spleen to identify different immune cells [17]. To better understand the cellular landscape of the catfish spleen we generated a snRNAseq atlas for the channel catfish. The spleen atlas provides a profile for the various immune cell types in the spleen and can be used to study the expression of immune-relevant gene families.

## Materials and methods

### Tissue preparation

This study was carried out at the United States Department of Agriculture–Agricultural Research Service (USDA-ARS) Aquatic Animal Health Research Unit (AAHRU) and the use of fish in this study was approved by the Institutional Animal Care and Use Committee (IACUC) to ensure ethical use of research animals. The protocol conformed to USDA-ARS Policies and Procedures 130.4 and 635.1. Three channel catfish (~ 1 kg) were obtained from an earthen production pond and euthanized in a solution of MS-222 (300 mg/L, Syndel USA, Ferndale, WA) buffered with sodium bicarbonate for 10 min. The individual spleens were removed and placed into Petri dishes containing channel catfish media (cL-15), which is made of Leibovitz’s L-15 medium (ThermoFisher Scientific, Waltham, MA) adjusted to catfish tonicity with PenStrep glutamine solution (ThermoFisher Scientific) and 5% FBS (R&D, Minneapolis, MN) [18]. The spleens were first processed through a 70 μM cell sieve into Petri dishes (ThermoFisher Scientific) to achieve a single cell suspension and then further processed through a 40 μM cell sieve (ThermoFisher Scientific) into 50 mL Falcon tubes and cL-15 was added up to 10 mL. The splenic cell suspensions were centrifuged at 350 x g for 5 min, the supernatant was aspirated, and the cell pellet was resuspended in 10 mL of cL-15. This process was repeated two additional times, and the final cell pellet was resuspended in 15 mL of cL-15. Splenic cells were counted, and cell viability was assessed with Trypan blue using a TC-20 cell counter (BioRad, Hercules, CA). Whole, unfractionated spleen preparations from each of the three individuals (1.0 x 10^7^ cells) were pelleted and resuspended in 1 mL of freezing medium (90% FBS, 10% DMSO) in 2 mL CRYO.S cryogenic vials (Greiner Bio-One, Monroe, NC) and placed into a Mr. Frosty freezing container (ThermoFisher Scientific) at -80°C for 24 h. The cryogenic vials were then moved into liquid nitrogen storage until shipping to a service provider.

### Cell preparation to isolate individual nuclei

The following procedures (isolation of nuclei through the generation of raw sequencing data) were performed via a service provider (SeqMatic, Fremont, CA). Frozen cells were thawed in a water bath set to 28°C and added to 6 mL of cL-15. The cells were centrifuged at 1200 RPM for 5 min to form a pellet. The supernatant was removed, and the cells were resuspended with 1 mL of cL-15. Cell concentrations and viability were measured using a Countess II FL automated cell counter (ThermoFisher Scientific). A total of 2.5 x 10^6^ cells were transferred to new tubes and centrifuged at 300 x g for 5 min at 4°C, and the supernatant was removed. The cells were lysed by gently pipette mixing in 200 μL of lysis buffer and incubated for 1 min on ice. The lysis buffer consisted of 10 mM Tris-HCL (pH 7.4), 10 mM NaCl, and 3 mM MgCl_2_ (Millipore-Sigma, Burlington, MA).

The lysed cells were initially washed by pipette mixing with 800 μL of Nuclei Wash and Resuspension Buffer and centrifuged. The buffer contained 1% BSA (ThermoFisher Scientific), 0.2 U/μL RNase Inhibitor (Millipore-Sigma), and 1x PBS (Corning-Cellgro). The nuclei were washed two times using 1 mL of Nuclei Wash and Resuspension Buffer, pelleted by centrifugation and the supernatant removed. Centrifugation for washing steps were carried out at 500 x g for 10 min at 4°C.

The quality of the nuclei was checked by resuspending with 100 μL of Nuclei Wash and Resuspension Buffer with Ethidium Homodimer-1 dye (Millipore-Sigma). The nuclei concentration was diluted to the target concentration required for library preparation.

### Library construction

The single-nuclei RNA-seq libraries were prepared and constructed using the Chromium X Instrument (10x Genomics, Pleasanton, CA) and the Chromium Next GEM Single Cell 3’ GEM Kit v3.1 (10x Genomics) following the manufacturer’s protocol. Briefly, the individual nuclei were partitioned to produce gel beads-in-emulsion (GEMs). The GEMs were generated by loading the nuclei in master mix, barcoded Single Cell 3’ v3.1 Gel Beads, and partitioning oil onto Chromium Next GEM Chip G. The Gel Beads was dissolved to release the primers and incubated to produce barcoded full-length cDNA. The cDNA was cleaned to removed left over reagents using Dynabeads MyOne SILANE (ThermoFisher), then amplified via PCR. Optimal sized cDNA amplicons were selected using SPRIselect (Beckman Coulter, Brea, CA) and Illumina indexes and adapters were added via end repair, A-tailing, adaptor ligation, and PCR. Sample quality was checked using a TapeStation (Agilent Technologies, Santa Clara, CA) with the TapeStation High Sensitivity D1000 ScreenTape (Agilent Technologies). The three cDNA samples were multiplexed and sequenced using the Illumina NovaSeq X Plus sequencer (San Diego, CA). The raw sequencing data was demultiplexed using Cell Ranger (v.5.0.0) `cellranger mkfastq`and data provided to the USDA-ARS-AAHRU.

### Data demultiplexing, trimming, filtering and normalization

Prior to snRNAseq data analyses, annotation of the channel catfish reference genome assembly (Coco_2.0, NCBI Accession number GCF_001660625.3) was manually curated. The annotation for IgM (ighm) was missing from the reference genome, and IgD (ighd) was annotated to include *ighm*. The reference genome annotation file (GCF_001660625.3_Coco_2.0_genomic.gtf) was adjusted to include an annotation for *ighm* (chr2:17077000–17085528) based on the channel catfish genomic DNA sequence of the IgM heavy chain (NCBI Accession number X52617.1), including four exons encoding CH1-4 and two exons encoding the transmembrane (TM) domains [19,20]. The annotation for *ighd* (NCBI Accession number XM_053677651.1) had exons (exon 1 and exon 2) that did not correspond with constant domains 1–7 or TM domains (NCBI Accession number AF363448) [10], and exon 2 overlapped with the *ighm* CH1 domain. Therefore, *ighd* was altered to the range chr2:17085529–17097451 and exon 1 and exon 2 removed.

A Cell Ranger compatible channel catfish reference genome was built using the reference assembly with the modified GTF file using `cellranger mkref`. Sequences were trimmed, aligned to the reference assembly, filtered, and counted to generate feature-barcode matrices.

The individual spleen samples were filtered further and normalized using Seurat (v.5.0.1) [21]. Cells with number of expressed genes < 200 and > 4,000, and cells that had expression from mitochondrial genes > 5% were removed from the data. The data was normalized using the global-scaling normalization method, whereby feature expression for each cell is normalized by the total expression, multiplied by 10,000 and log transformed.

### Cluster analysis

The dimensionality of the data was analyzed by principal component analysis (PCA). Prior to PCA, the expression data was scaled so that the mean expression across cells was 0, and the variance was 1. An elbow plot of the PCA standard deviations and observation of genes in PCAs was used to determine the number of dimensions used to generate clusters.

Cluster analysis was carried out on individual samples and on integrated (aggregated) samples. Samples were integrated using the `merge`, `integrate`and `join`functions in Seurat. Clusters were generated through a graph-based approach using the `FindNeighbors`and `FindClusters`functions in Seurat. Briefly, a K-nearest neighbor (KNN) graphing approach identified cells with similar gene expression patterns, and the Louvain algorithm was applied to identify clusters. The clusters were labelled as erythroid, hematopoietic stem cells (HSC), B cells, T/NK cells, myeloid-derived cells or endothelial cells depending on gene marker expression. Markers used to define cell type were obtained from the current literature. Differential gene expression analysis was carried out between clusters using `FindAllMarkers`.

### Cell trajectory analysis

The trajectory analysis was conducted using Monocle 3 (v1.3.7) [22]. The Seurat object containing cluster information was converted to a cell data set object using SeuratWrappers (v0.3.5). The function `learn_graph`was used to predict the trajectory, and `order_cells`was used to determine the pseudotime of each cell. The trajectory and pseudotime were plotted onto the UMAP of clusters.

## Results and discussion

### Data quality

The snRNAseq libraries of each spleen from three individual channel catfish were termed SP1, SP2, and SP3. The summary statistics of the splenic snRNAseq transcriptome libraries, before filtering, generated an average of 278,717,872 reads from a mean 8,157 cells (Table 1). Filtering was carried out to remove cells likely to be non-viable, identified by higher mitochondrial DNA counts (> 5%), and from cells likely to be doublet/multiplet indicated by high molecule/feature counts. After filtering, 81.4%, 69.6% and 84.0% of cells remained for SP1, SP2, and SP3, respectively.

**Table 1:** Summary statistics of single-nuclei transcriptome libraries (SP1, SP2, SP3) generated from catfish spleens (n = 3). The data were initially filtered to remove barcodes not associated with cells using Cell Ranger and further filtering was carried out using Seurat to remove multiplets and cells with > 5% reads aligned to mitochondrial genes. The individual libraries were integrated for combined cluster analysis in Seurat.

Sample	Output	SP1	SP2	SP3	Seurat Integrated Samples
Number of Reads	Cell Ranger ^a^	198,304,083	215,231,958	422,617,575	
Median Number of Reads/Cell	Cell Ranger ^a^	25,591	42,037	36,423	
Q30 Bases in RNA Reads	Cell Ranger ^a^	95.0%	93.6%	95.2%	
Confident Alignment to Reference Transcriptome	Cell Ranger ^a^	48.4%	47.7%	42.1%	
Estimated Number of Cells	Cell Ranger ^a^	7,749	5,120	11,603	
	Seurat ^b^	6,305	3,564	9,744	19,613
Number of Features Detected	Cell Ranger ^a^	25,591	22,692	23,1456	
	Seurat ^b^	19,094	19,224	20,121	20,121
Median Number of Features/Cell	Cell Ranger ^a^	647	554	733	
	Seurat ^b^	649	532	737	665

^a^ The data were initially filtered to remove barcodes not associated with cells using Cell Ranger.

^b^ The data after filtering using Seurat.

**Fig 1:**
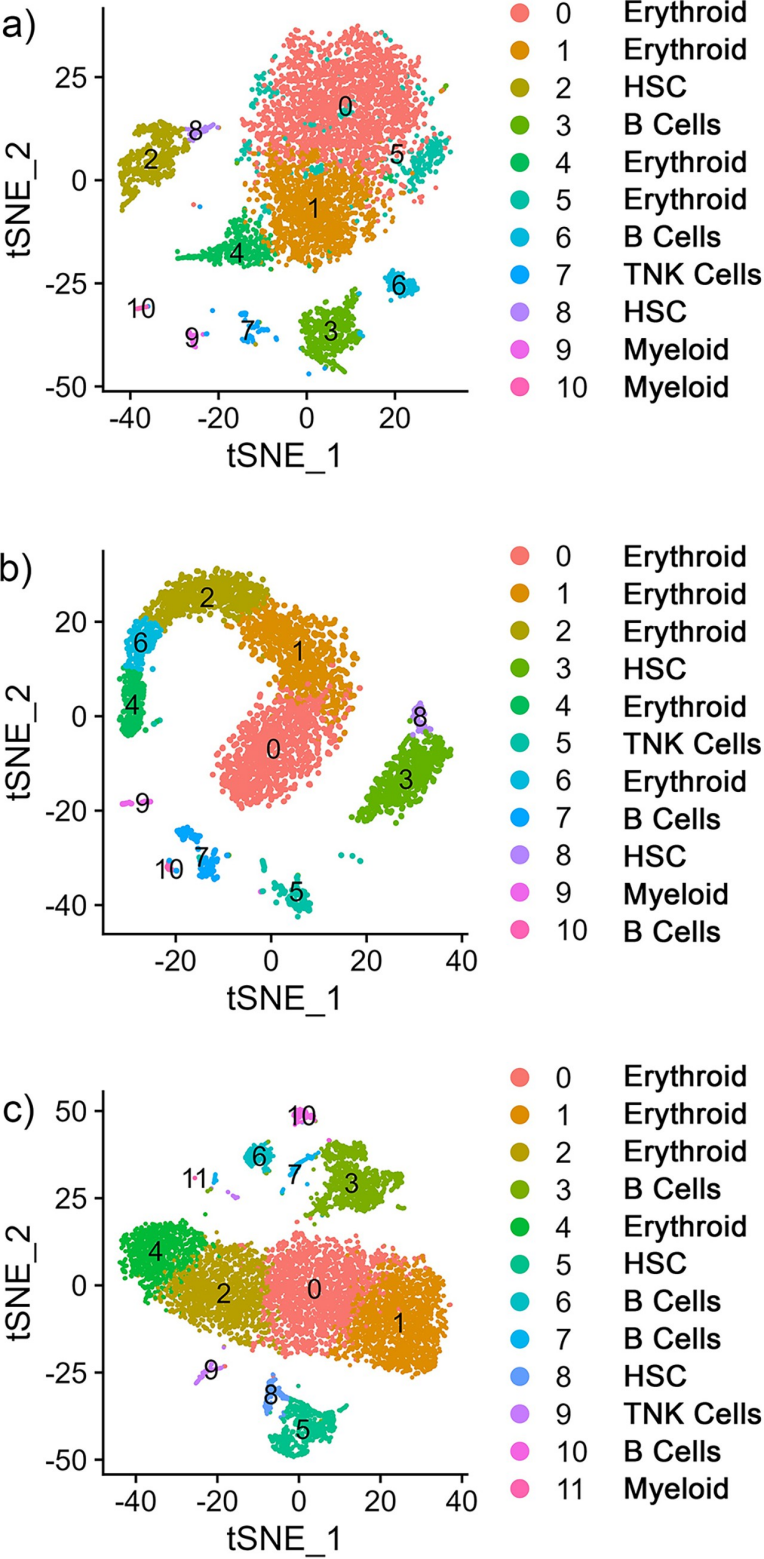
Single-nuclei transcriptomes of whole spleen samples from channel catfish (*Ictalurus punctatus*). Cluster analysis was performed on individual samples (a) SP1 (b) SP2 (c) SP3. Clusters are depicted as tSNE plots and cell-markers are used to classify clusters as erythroid cells, HSC, B cells, T/NK cells, and myeloid lineage cells.

The individual cluster analyses identified 11 to 12 clusters for SP1, SP2, and SP3, which are numbered sequentially in order of cell number in each cluster per individual (Fig 1). The numbering does not necessarily indicate that clusters with the identical numbering between samples are the same cell type. The ten most differentially expressed gene markers were identified for individual clusters (S1 Table). Cell clusters for each library were defined when a similar number of clusters, and cluster-size distribution were identified, and an initial cell identification was proposed based on the expression of canonical genes from known cell types (Fig 1 and S1 Table). To identify cell types within the spleen libraries, genes identified from single-cell analyses of different vertebrate species (mammalian and teleost) were used as a reference (Table 2). Ultimately, the individual sample libraries were integrated into one dataset for cluster analysis and cell type identification. Except for one cluster, cells from all three samples contributed to the clusters in the integrated single-cell data set (S1 Fig).

**Table 2:** Features used to identify each cell type among different clusters.

Population	Feature	Reference
Erythroid cells	*slc4a1a*, *alas2*, *sptb*, *hemgn*	[6,23,24]
B cells	*pax5*, *cd79b*, *cd37*, *ighd*, *ighm*	[6,25]
T/NK cells	*lck*, *runx3*, *ifgn1*, *il7r*	[6,26,27]
HSC	*meis1b*, *mpl*, *runx1*, *gata2b*	[6,28–30]
Myeloid cells	*adgrg3*, *nccrp1*, *mpeg1*.*1*, *grna*	[6,31]
Endothelial cells	*cdh5*, *kdrl*, *pecam1a*, *akap12b*	[6,32]

### Cell populations in the channel catfish spleen

**Fig 2:**
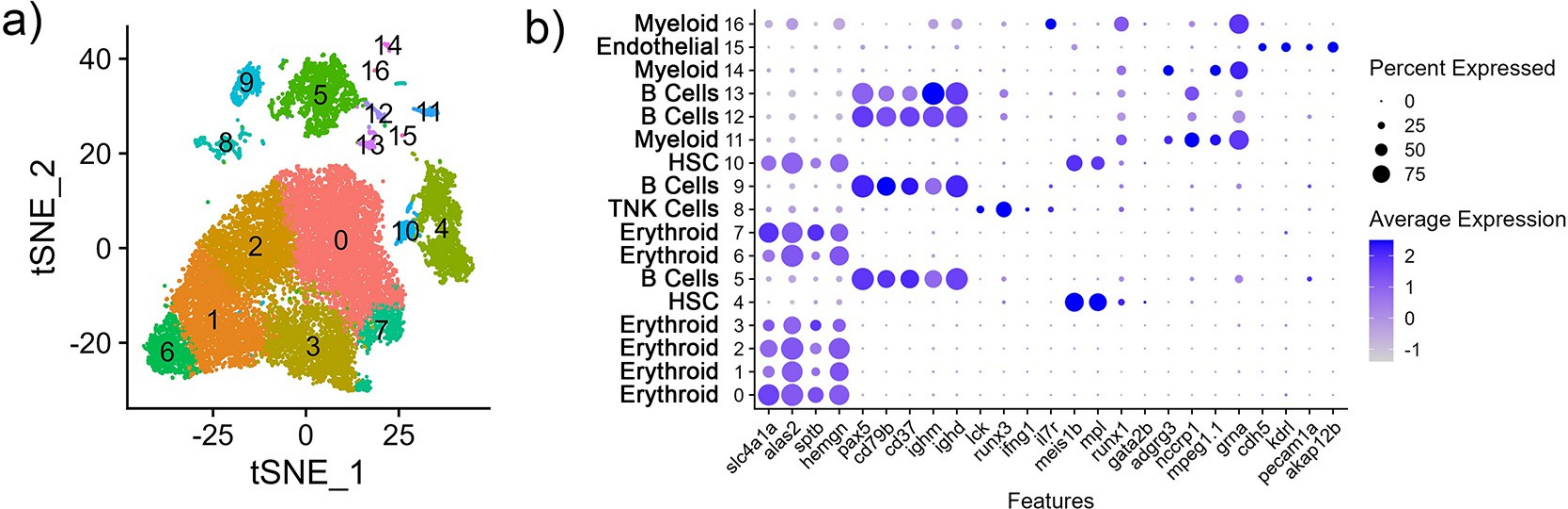
Cluster analysis of integrated nuclei transcriptome of spleen (n = 3) from channel catfish. Cluster analysis identified sixteen cell clusters. (a) tSNE plot (b) Average expression (z-score) of cell type markers (x-axis) for each cluster (y-axis). The size of the dot represents the percentage of cells per cluster contributing to expression. Cell type was assigned to clusters based on the expression of canonical cell markers (Table 2).

Cluster analysis of the aggregated spleen library yielded 17 clusters which were defined in the tSNE (Fig 2A) and heat map (S2 Fig). Expression of 25 canonical gene markers defined these different clusters (Fig 2B and Table 2). The majority of identified cells (14,353; 73.0%), consisting of six clusters, expressed erythroid markers. The immune cell markers further defined hematopoietic stem cells (HSC, 2,065; 10.5%), B cells (2,469; 12.5%), T/Natural Killer (NK) cells (461; 2.35%) and myeloid lineage cells (234; 1.19%). Thirty-one cells expressing endothelial markers were also identified (S2 Table and S2 Fig).

The six erythroid clusters 0–3 and 6–7 had increased expression of *slc4a1a*, *alas2*, *sptb* and *hemgn*, and uniformly grouped together (Fig 2 and S2 Table). Cluster 0 had significantly increased expression of blood related genes *LOC108257039* (hemoglobin subunit beta), *blvrb* (biliverdin reductase B), *hbaa2* (hemoglobin subunit alpha 2), *mt2* (metallothionein 2), and *urod* (uroporphyrinogen decarboxylase). In humans, *MT2A* is a negative regulator of erythroid differentiation [33]. The human orthologue of *blvrb* maintains redox homeostasis and is involved in directing cell fate of HSC to red blood cells [34]. The gene *urod* encodes an enzyme involved in heme production [35]. Cluster 1 has significantly increased expression of *pcmt* (protein-L-isoaspartate O-methyltransferase) which is involved in repair of red blood cells undergoing oxidative stress in mice [36]. Therefore, this cluster may represent red blood cells with oxidative stress. Cluster 2 cells significantly increased expression of hemoglobin subunits, *LOC108257036* (hemoglobin subunit beta) and *LOC108257041* (hemoglobin subunit alpha). The remaining genes highly expressed by this cluster have not been characterized. Cluster 3 cells have increased expression of *ank1a* (ankyrin 1), *sox6* (SRY-box transcription factor 6), *LOC108280488* (transferrin receptor), *ripor3*, *slc7a5* (Solute carrier family 7 member 5). Ankyrin is a protein that attaches to the cell membrane to maintain the membrane structure of erythrocytes [37]. *Sox6* is involved in erythroid maturation [38]. Human and murine orthologues of *slc7a5* control red blood cell size and maturation and absent in mature erythroid cells [39]. Therefore, Cluster 3 cells may represent immature erythroid cells. Cells in cluster Erythroid 6 was defined only by increased ribosomal protein expression. Cluster 7 had increased expression of *slc4a1a*, *LOC108280488* (transferrin receptor), *ripor3*, *ank1a*, *sptb*, *blvrb* and *epb41b* (erythrocyte membrane protein band 4.1b).

Cluster 4 had increased expression of *itga2b* (integrin alpha 2b; also known as *cd41*), *meis1b* (meis homeobox 1) and *itgb3b* (integrin beta 3b; also known as *cd61*) which are genes known to be expressed by HSC (Table 2). *Itga2b/itgb3b* are used as a marker for HSC in teleost fish [40,41] and other species [42–44]. The expression of the transcription factor *meis1b* has been detected in HSC isolated from zebrafish kidney [45], and was shown to be important for the maintenance of adult mouse HSC [46]. Cluster 10 (HSC) expressed both erythroid (*slca1a*, *alas2*, *sptb*, *hemgn*) and HSC markers (*meis1b*, *mpl*, *runx1*).

There are four clusters (Clusters 5, 9,12–13) that have expressed B cell markers *pax5*, *ighd*, *ighm*, *cd79b* and *cd37* (Fig 2B and S2 Table). Clusters 5 and 9 have *pax5*, *ighd*, *LOC108259908* and *nr4a1* (nuclear receptor subfamily 4 group A member 1) among their most highly expressed genes. *LOC108259908* is assigned to an Ig heavy chain Mem5-like gene in the current channel catfish genome assembly (Coco_2.0), but likely represents an IgL gene segment based on BLAST analysis [47,48]. *Pax5* and *ighd* are canonical B cell genes [49,50] and *nr4a1* is expressed in a wide variety of immune cells, including macrophages, but, in mice, functions to restrain immunodominance of B and T cells when diverse clonal population compete in the germinal center (GC) [51,52]. In the apparent absence of GC in teleost fish perhaps it is more important in regulating the formation or maintenance of melano-macrophage centers [53,54]. Cluster 5 also had increased expression of *ebf1b* (early B cell transcription factor 1b), *LOC108261469* (immunoglobulin kappa constant-like) and *pou2af1* (POU class 2 homeobox associating factor 1). In teleost, *ebf1b* is expressed by the common lymphoid progenitor, pro-B cells and pre-B cells and drives early B cell commitment [49]. This suggests that the cells in Cluster 5 could represent a nascent B cell lineage. Cluster 9 also had increased expression of *LOC108267808* (LYN proto-oncogene) and *cd79b*. Expression of *LYN* and *cd79* have been shown to be important for B cell receptor formation, maturation, and survival [55,56].

The remaining B cell clusters, 12 and 13, have higher expression of *fabp3* (fatty acid binding protein 3) and *mki67* (marker of proliferation Ki-67). In mice, *fabp3* is expressed in activated B cells and promotes the development of plasma cells [57]. *MKI67* expression was increased in human bone marrow plasmablasts [58]. The expression of *fabp3* and *mki67* indicates that these cells may have differentiated from B cells to plasmablasts. The cells in Cluster 12 also have increased expression of *diaph3* (diaphanous-related formin 3) and *atp5mc1* (ATP synthase membrane subunit c locus 1). The human orthologues of *diaph3* and *atp5mc1* are expressed by human plasma cells [59]. The cells in Cluster 13 have increased expression of *pdia4* (protein disulfide isomerase family A, member 4), *selenom* (selenoprotein M), *spats2* (spermatogenesis associated serine rich 2), *hsp90b1* (heat shock protein 90 beta family member 1), *stt3a* (STT3 oligosaccharyltransferase complex catalytic subunit A), *ube2j1* (ubiquitin conjugating enzyme E2 J1), and *lman1* (Lectin, mannose binding 1). In mice, it has been shown that *hsp90b1* acts as a chaperone to toll-like receptors and integrin molecules [60]. The human single cell type atlas indicates that *PDIA4*, *SELENOM*, *SPATS2*, *HSP90B1*, *STT3*, *UBE2J1* and *LMAN1* are expressed by human plasma cells [59].

Cluster 8 expressed T cell markers *lck*, *runx3*, *ifng1* and *il7r* (Fig 2B and S2 Table). The most differentially expressed genes indicative of T cell function include *ptprc* (protein tyrosine phosphatase receptor type C), *runx3*, *fmnl1a* (formin like 1) and *arid5a* (AT-rich interaction domain 5A). *Ptprc* is expressed by most immune cells but is important for T cell development and signaling, and NK cell function [61–63]. *Runx3* is a transcription factor for T cell development [64]. In mice, *fmnl* was shown to be important for T cell migration through restrictive environments [65]. *Arid5a* directs differentiation of naive CD4^+^ T cells to inflammatory CD4^+^ T cells [66].

Clusters 11, 14 and 16 have increased expression of *adgrg3*, *nccrp1*, *mpeg1*.*1* and *grna* indicating that they likely belong to the common myeloid cell lineage (Fig 2B). Cluster 11 was defined by upregulated expression of myeloid-related genes such as *dbn1* (drebrin 1), *foxp4* (forkhead box P4), *samsn1a* (SAM domain, SH3 domain and nuclear localization signals 1), *csf1rb* (colony stimulating factor 1 receptor), *mafba* (MAF bZIP transcription factor B) and *csf3r* (colony stimulating factor 3 receptor). *Dbn1* is expressed by DCs and mast cells in mice and encodes drebrin which is an actin binding and stabilizing protein [67,68]. An *in vitro* study of *FOXP4* has shown that it may have a role in the inflammatory response of human neutrophils [69]. The *samsn1* mouse orthologue regulates inflammatory response of immune cells, including macrophages, mast cells and lymphoid cells [70]. In humans, *MAFB*, a transcription factor, is involved in cell fate of myeloid cells which can differentiate to either macrophages and DCs [71]. Lastly, *csf1r* encodes a receptor that regulates monocytes and macrophages in teleost species [72], and was demonstrated to promote the differentiation of myeloid progenitors into leukocytes such as monocytes, macrophages and DCs in human and mouse studies [73]. *LOC108266645* (killer cell lectin-like receptor subfamily E member 1) is also highly expressed and may indicate pre-cursors to DCs are present in this group. Killer cell lectin-like receptors typically modulate DC and NK cell immune responses [74,75].

Cluster 14 had increased expression of myeloid-lineage genes *flt3* (fms related receptor tyrosine kinase 3), *fgd4a* (FYVE, RhoGEF and PH domain containing 4a), *plxna4* (plexin A4), *dbn1*, *ppfibp2* (ppfibp2a), *pak1* (P21 (RAC1) activated kinase 1). *Flt3* is involved in differentiation of myeloid progenitors into macrophages (mouse) and DCs [76,77]. The *pak1* mouse orthologue was shown to be involved with macrophage polarization [78]. Human orthologues of *fgd4a* and *ppfibp2* have expression in monocytes, and *plxna4* has expression in DCs. Myeloid related genes that were upregulated in Cluster 16 were *apol1* (apolipoprotein L1), *sting1* (stimulator of interferon response cGAMP interactor 1), *LOC108279184* (transcription factor 4; *tcf4*), *LOC108266986* (TLR adaptor interacting with endolysosomal SLC15A4), *nitr6* (novel immune-type receptor 6) and *ctss1* (cathepsin S). There is some evidence that human *apol1* orthologues may be involved with DC cell death [79]. *Sting1* is a well-characterized transmembrane protein of the endoplasmic reticulum involved in inflammation and immune response to pathogens by producing type 1 interferons and pro-inflammatory cytokines [80]. Human *STING1* expression is detected in diverse immune cells, including macrophages, T cells and NK cells [59]. The human orthologue of *LOC108266986*, *TASL*, is involved in immune responses through the production of cytokines and chemokines, primarily in plasma cells, DCs and macrophages [81]. The human orthologue of *LOC108279184*, *TCF4*, has been shown to be expressed by human DCs [59] and important for plasmacytoid DC development [82]. *Nitr* family genes were first identified in pufferfish and do not have a human orthologue [83]. This family of genes are predicted to have similar functions to leukocyte receptor complex genes [84]. *Ctss1* encodes cathepsin S and reported to be expressed by macrophages, B cells and dendritic cells [85]. In *I*. *punctatus*, cathepsin S genes were upregulated in mucosal surfaces in response to bacterial infection [85].

Cluster 15 expressed endothelial marker genes *cdh5*, *kdrl*, *pecam1a*, *akap12b*, *znf521*, *calcrl*. These cells are most often associated with vascular and lymphatic development [86,87].

### Characterization of the immunome

To gain additional insight into the splenic immunome, the erythroid cells (Fig 2) were filtered from the integrated data and cluster analysis was again performed on the remaining 5,269 cells. This analysis identified 15 immune cell clusters (S3 and S4 Figs and S3 Table).

Additional subclusters have been defined for HSC, B cells, T cells and myeloid cells. The archetype markers (Table 2) were used to validate the subcluster cell types and differential expression analysis identified the unique expression patterns of each subcluster.

**Fig 3:**
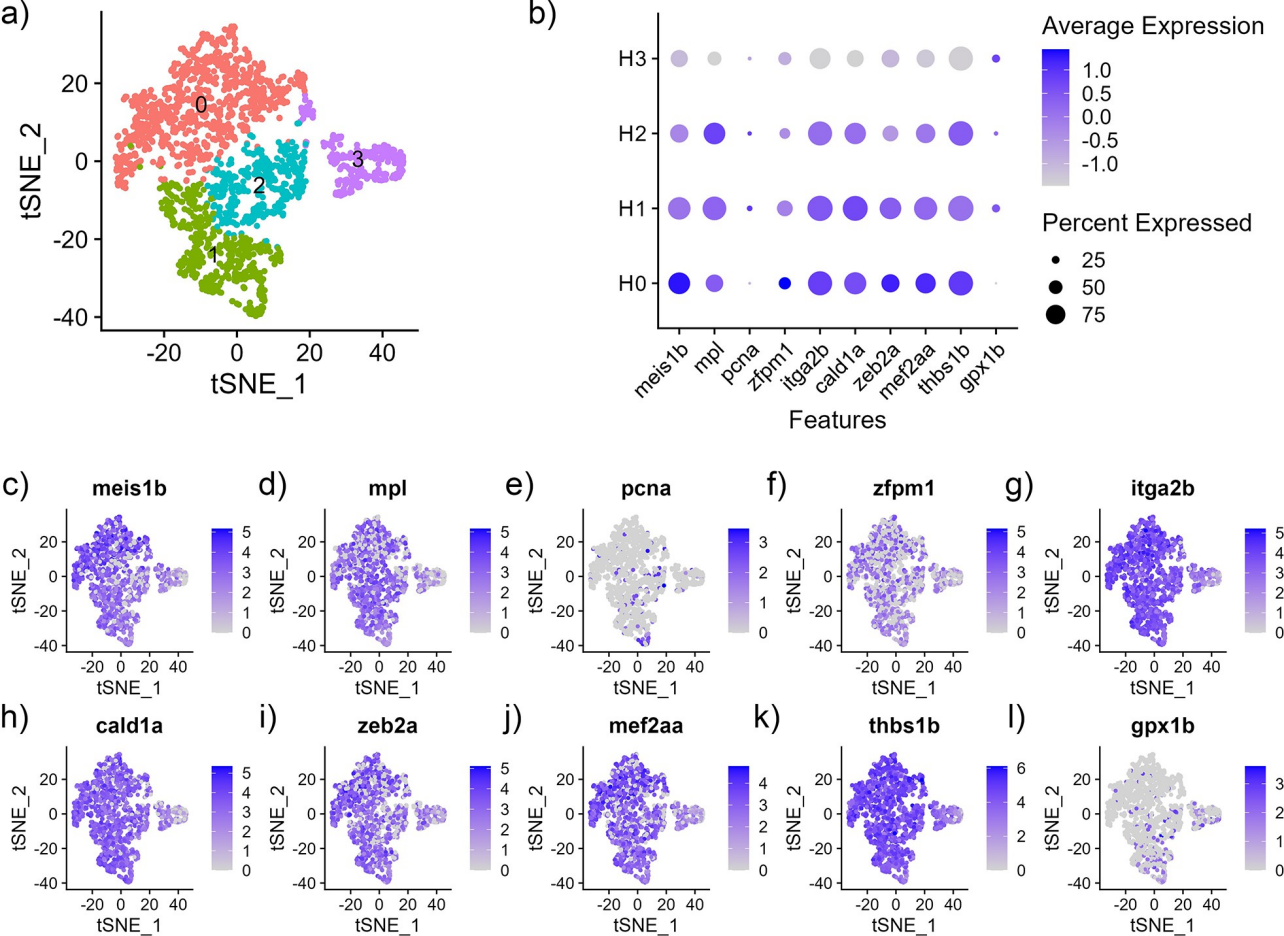
Subcluster analysis of hematopoietic (HSC) cells from the integrated nuclei transcriptome of spleen from channel catfish. (a) tSNE plot (b) Average expression (z-score) of cell type markers (x-axis) for each cluster (y-axis). The size of the dot represents the percentage of cells per cluster contributing to expression. (c-l) Feature maps of HSC gene expression on tSNE plots.

HSC cells were further defined into 4 subclusters, HSC0 (932 cells), HSC1 (483 cells), HSC2 (371 cells), HSC3 (279 cells) using the top differentially expressed genes (Figs 3A and S5, S4 Table). HSC0-2 seemed consistent in their expression of known HSC genes (Fig 3B–3L) [40,41]. HSC3 however had much less expression of these genes and an increased expression of genes often associated with erythroid-cell function such as *alas2* (5’-aminolevulinate synthase 2), *hbb* (hemoglobin subunit beta), *slc4a1a* (solute carrier family 4 member 1), and *LOC108257040* (hemoglobin subunit alpha) (S4 Table). This cluster also had increased expression of the erythroid markers *sptb* and *hemgn*. One hypothesis is that the HSC that express erythroid markers are erythroid progenitors. Hematopoietic stem and progenitor cells with erythroid marker expression have also been identified in the zebrafish spleen [6]. The authors suggested that these cells were potentially erythroid progenitors. Another hypothesis is that the erythroid markers represent partially differentiated cells that can develop into multiple cell lineages. The erythroid markers were common between three subclusters of cells for HSC, B cells, and T cells. Thus, since HSC differentiates to lymphocytes, the cells could be progenitors. A third hypothesis is that the erythroid-like cells are erythrocytes with immune functions. There is evidence of erythrocytes expressing some immune genes. In the case of Nile tilapia (*Oreochromis niloticus*), erythrocytes expressed interferon regulatory factors that were also expressed by leukocytes [88]. However, our data shows that the channel catfish erythroid-like cells have similarities with lymphocytes. Additional investigation is required to further elucidate the classification of these cell subtypes.

**Fig 4:**
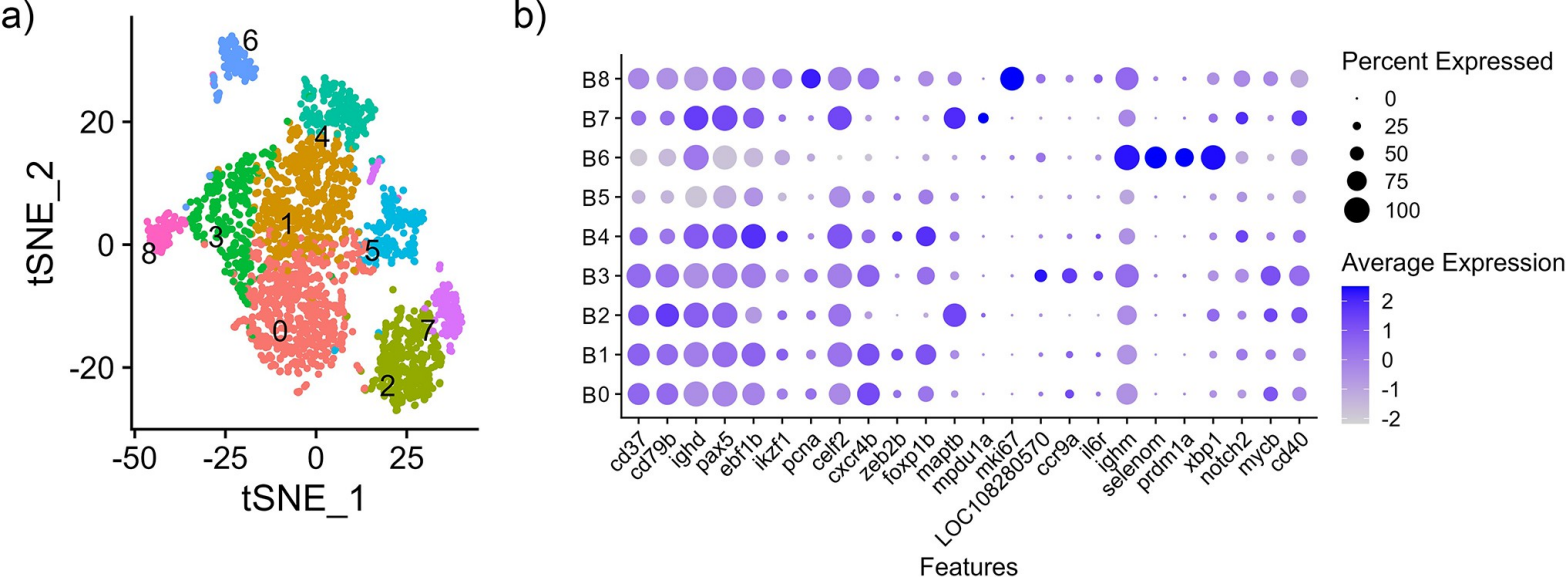
Subcluster analysis of B cells from the integrated nuclei transcriptome of spleen from channel catfish. (a) tSNE plot (b) Average expression (z-score) of cell type markers (x-axis) for each cluster (y-axis). The size of the dot represents the percentage of cells per cluster contributing to expression.

Further examination of 2,469 splenic B cells identified 9 subclusters, B0-B8 (Fig 4A), wherein these subclusters have expressed the canonical B cell markers and further been defined using the top differentially expressed genes (Figs 4B and S6, S5 Table). Based on their gene expression we sought to identify different B cell lineage developmental stages [89,90]. In subcluster B1 (489 cells), *zeb2b* was most significantly expressed and in subcluster B4 (201 cells), *ebf1b* was the most significant (S5 Table). Subclusters B1 and B4 also both had higher expression of *ikzf1* and *foxp1b* relative to the remaining cluster cells (Figs 4B and S7). These genes are well defined transcription factors required for early B cell differentiation [91,92]. Subcluster B1 also had significantly highly expressed *ets1*, another known B cell development regulator [93]. Taken together these results suggest that B1 and B4 likely represent an immature stage of B cell development in the spleen. In contrast, subclusters B2 (343 cells), B3 (288 cells) and B7 (124 cells) relative to the remaining clusters had higher expression of *il6r* and *cd40*, cell surface receptors, and *mycb*, a transcription factor [94,95]. B3 also had higher expression of *cxcr4b* and the analysis of the most differentially expressed genes identified *LOC108280570* (*homer3*) and *ccr9a* expression (S5 Table). Therefore, B2, B3 and B7 likely represent varying mature B cell populations. Lastly, subclusters B6 (125 cells) and B8 (103 cells) have higher expression of genes associated with plasmablasts and plasma cells (Fig 4B). B6 has greater expression of *selenom* and *irf4l* (S5 Table), and relative to the other clusters had higher expression of *ighm*, *prdm1a* and *xbp1* (Figs 4B and S7) [96–98]. B8 had significantly greater expression of *mki67* and *top2a* (S5 Table) and relative to some clusters had less expression of *pax5* (Figs 4B and S7).

**Fig 5:**
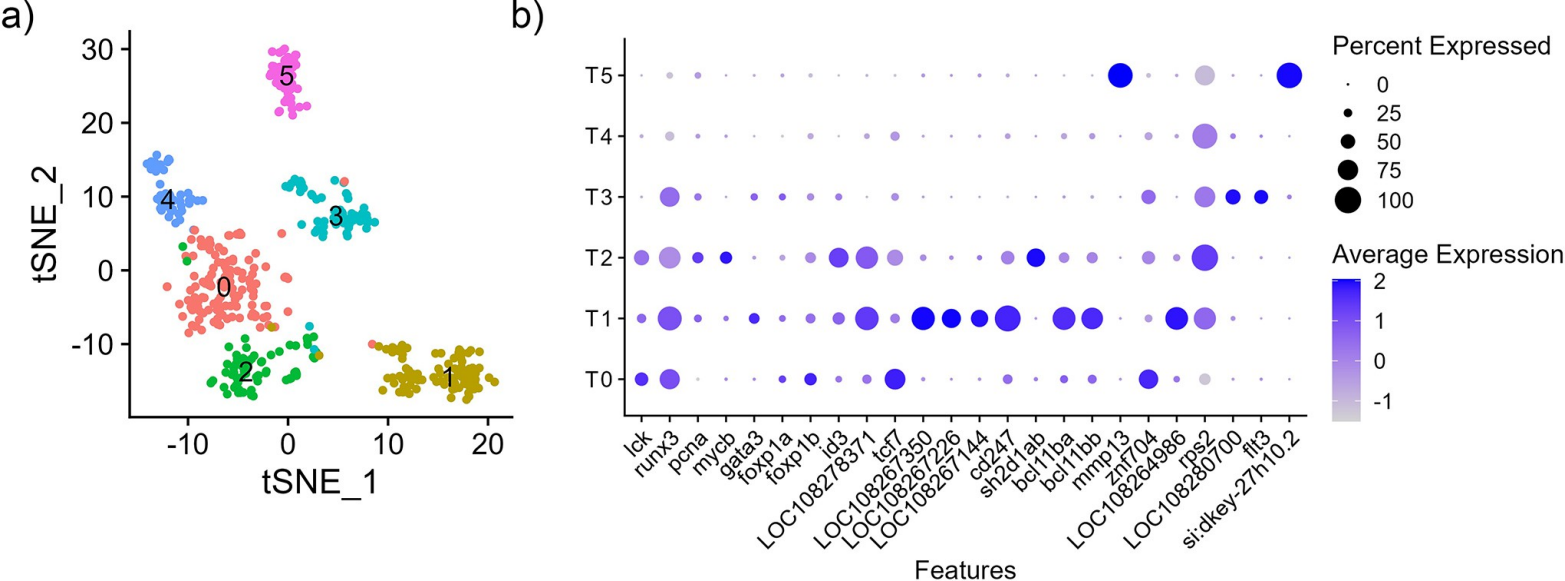
Subcluster analysis of T/NK cells from integrated nuclei transcriptome of spleen from channel catfish. (a) tSNE plot (b) Average expression (z-score) of cell type markers (x-axis) for each cluster (y-axis). The size of the dot represents the percentage of cells per cluster contributing to expression.

Cluster analysis using 461 splenic T/NK cells identified 6 subclusters which have expressed canonical T and NK cell markers and were further defined using the top differentially expressed genes (Fig 5A, S6 Table and S8 Fig). Interestingly, except for a few cells, the T cells did not express *cd4* (*cd4-1*, *cd4-2*.*2*) or *cd8* (*cd8a*, *cd8b*) genes (S9 Fig) suggesting that these cells are not differentiated as helper T cells or cytotoxic T cells. Subcluster T0 (127 cells) most significantly expressed *tcf7*, *LOC108256553* (DNA-binding protein SATB1), and *rgs3a* (regulator of G protein signaling 3a) (Fig 5B and S6 Table), which facilitate differentiation and regulation of T cell function [99,100]. T0 also had significantly increased expression of *tox* (thymocyte selection-associated high mobility group box) and *tox2* (TOX high mobility group box family member 2) that also contribute to the functional maintenance of T cells and NK cells (S9 Fig and S6 Table) [101–107]. T0 also has significantly expressed transcription factors *LOC128635459* (*znf239*) and *znf704*, which also likely have a role in lymphocyte development and are representative of naïve T cells [108]. Subcluster T1 (91 cells) has significantly increased expression of several genes that make up the T cell receptor complex (TCR). These genes include gamma (*LOC108267226*, *LOC108267144*), delta (*LOC108267350*), and *cd247* TCR genes (Fig 5B and S6 Table) [109,110]. T1 also had increased expression of *LOC108264986* (tyrosine-protein kinase ZAP-70*)*, a gene that encodes a tyrosine kinase involved in the proximal TCR signaling and T cell activation [111], *mafa* (MAF bZIP transcription factor), which is a transcription factor that regulates T cell specification and maintenance [112] and *bcl11ba*, a zinc finger transcription factor that is critical for T cell maintenance and identity [113]. Subcluster T1 expression seems to be consistent with that of γδ T cells; however, they are not often associated with primary lymphoid tissues in vertebrates [114,115]. Subcluster T2 (72 cells) had significant expression of *sh2d1a* (SH2 domain containing 1A), *hcst* (hematopoietic cell signal transducer; also known as *DAP10*), *gzmb3*.*3* (granzyme B) and *gzmk* (granzyme K), that have functional pathways in both T and NK cells (Figs 5B and S9, S6 Table) [116–119]. T2, relative to the other subclusters, also had higher expression of *id3* and *LOC1082788371* (*CD3ε*) (Fig 5B) which, taken together, would likely classify these as effector T cells [120]. Subcluster T3 (65 cells) had increased expression of genes that encode receptors and membrane bound proteins found expressed among different immune cell types, thus, the cells may represent innate-like lymphoid cells (ILC). T3 subcluster genes, *flt3* (fms related receptor tyrosine kinase 1), *LOC108266645* (killer cell lectin-like receptor subfamily E member 1), *prf1* (perforin 1) and *pag1* (phosphoprotein membrane anchor with glycosphingolipid microdomains 1) represent functional pathways in DC, NK and T cell phenotypes. In addition, *zeb2b* (zinc finger E-box binding homeobox 2) also has non-specific immune cell expression [59]. Subcluster T4 is characterized by greater expression of erythroid genes, including *LOC108257039* (hemoglobin cathodic subunit beta), *alas2*, *LOC108268628* (calpain8), *gpx1a*, *blvrb*, *hba*, and *cahz*. Interestingly, subcluster T5 (53 cells) had increased expression of myeloid markers. Four of the top ten differentially expressed genes, *mmp13* (matrix metallopeptidase 13), *mmp9* (matrix metallopeptidase 9), *ms4a4a* (membrane spanning 4-domains A4A) and *gpr84* (G protein-coupled receptor 84), represent functional processes in macrophages [121–124]. Furthermore, human macrophages express *TKT* (transketolase; *LOC108265455* in channel catfish), *CUX1* (cut like homeobox 1; *si*:*dkey-27h10*.*2* in channel catfish), *MMP9*, *MS4A4A*, *FBP1* (Fructose-bisphosphatase 1) and *GPR84* [59]. Only two of the top 10 genes, *LOC108265455* (transketolase) and *gpr84* have known functions in T cells [125,126]. Thus, the T5 subcluster has an indeterminate cell type with macrophage-like expression patterns (Figs 5B and S8).

**Fig 6:**
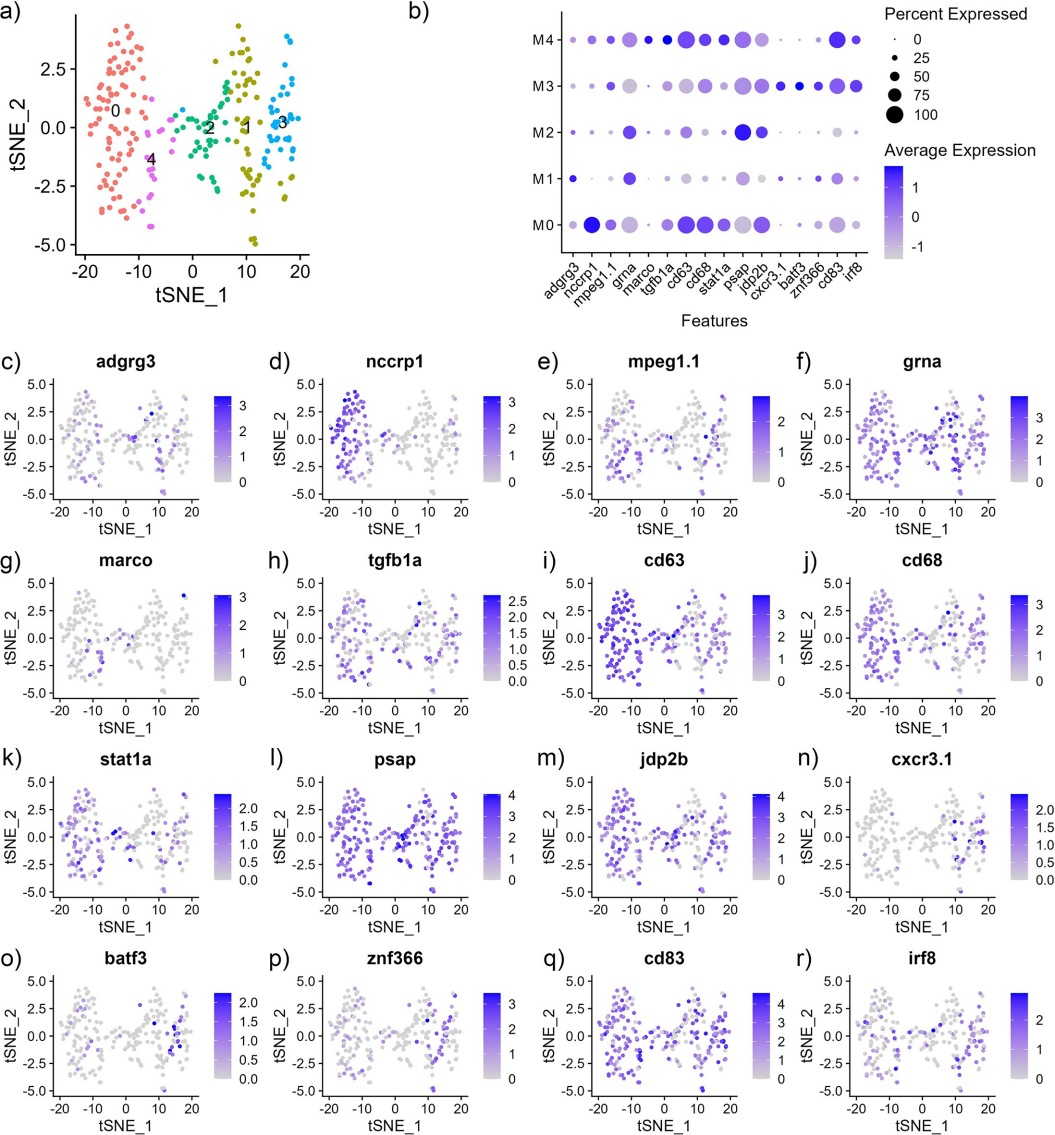
Subcluster analysis of myeloid cells from integrated nuclei transcriptome of spleen from channel catfish. (a) tSNE plot (b) Average expression (z-score) of cell type markers (x-axis) for each cluster (y-axis). The size of the dot represents the percentage of cells per cluster contributing to expression.

Subcluster analysis was performed on the 234 myeloid cells, and five unique groups of cells with distinct expression patterns in the heat map (Figs 6A and S10) and differentially expressed genes (S7 Table) were identified. After scrutiny of differentially expressed genes, two DC clusters and three macrophage subclusters were identified. M0 subcluster (81 cells) significantly expressed the catfish gene *si*:*dkey-5n18*.*1*, an ortholog of human *C1QL3* (complement C1q like 3), which comes from the same gene family as *C1Q* (complement C1q). *C1Q* expression in macrophages has been implicated in the functional process of eliminating apoptotic cells [127]. The cells in the M1 subcluster (52 cells) were putatively identified as DCs as they had increased expression of *flt3* (fms related receptor tyrosine kinase 3) and *irf4a* (interferon regulatory factor 4), which have DC functions [128,129]. Subcluster M2 (40 cells) had increased expression of *lrp1ab* (low density lipoprotein receptor-related protein 1Ab) which has a function in macrophages [130]. M2 also has increased expression of *rgl1* (ral guanine nucleotide dissociation stimulator-like 1) and *LOC108277878* (sialoadhesin). The human orthologs of these genes are expressed in macrophages [59]. The subcluster M3 (39 cells) had greater expression of *cd74b and cytip* (cytohesin 1 interacting protein) which have functions in DCs [131] and is also highly expressed in human monocytes [59]. The M3 cells also expressed genes typically transcribed by DCs, *cxcr3*.*1* (C-X-C motif chemokine receptor 3), *batf3* (basic leucine zipper ATF-like transcription factor 3), and *znf366* (zinc finger protein 366) (Fig 6B). The subcluster M4 (22 cells) had increased expression of macrophage genes *marco*, *mafba* (MAF bZIP transcription factor B), *csf1r* (colony stimulating factor 1 receptor), *csf1* (colony stimulating factor 1), *c1qb* (complement C1q B chain) and *c1qc* (complement C1q subcomponent subunit C) [71,72,132–136].

### Cell trajectory and pseudotime analysis

**Fig 7:**
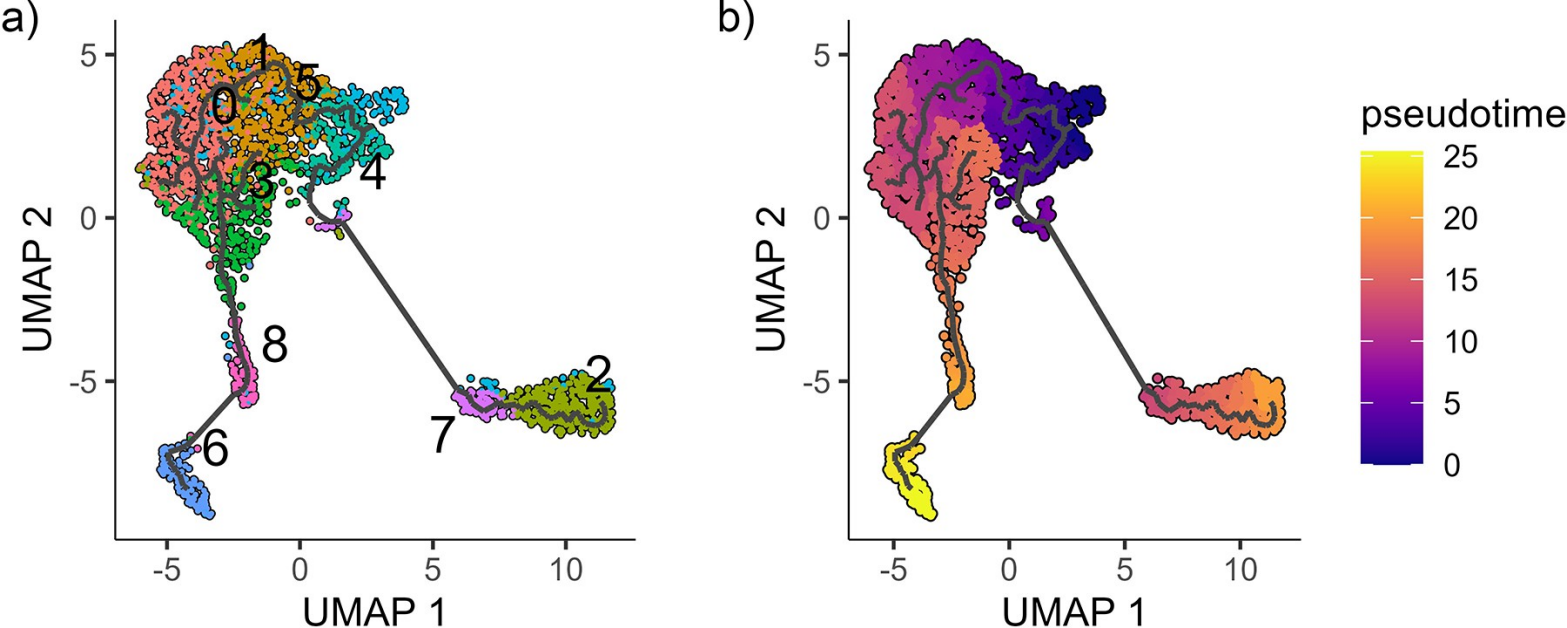
Trajectory analysis of B cells from integrated nuclei transcriptome of spleen from channel catfish (n = 3). (a) Predicted B cell trajectory branching and (b) predicted pseudotime determined by Monocle 3.

The subcluster analysis of B cells suggested that there were different stages of B cell lineage in the spleen, including immature B cells to plasma cells. The analysis with Monocle 3 was used to determine a trajectory path between clusters by using the Louvain community detection algorithm to group mutually similar cells, and then merging adjacent groups into ‘supergroups’. Using this information, Monocle 3 then predicts a path that cells can take and identify branches within supergroups. The trajectory path between the B cells was projected onto the UMAP produced using Seurat (Fig 7A). Pseudotime was then calculated using nodes within subcluster B4 as root cells (Fig 7B). Cells in B4 had the higher expressions of *ebf1b*, *ikzf1* and *pax5* which suggested that they are immature B cells, and the earliest developmental time-point. The trajectory and pseudotime predict that the immature B cells can branch towards two populations (Fig 7A). One path shows a trajectory toward the mature B cells and then plasmablasts (B8) and plasma cells (B6). While, another branch connects the root cluster, B4, to subclusters B7 and B2. The B cells from B7 and B2 appear to have greater differences to the other subclusters, although, their identity is not known. These subclusters may represent circulating B cells that are not resident in the spleen.

## Conclusion

Single-cell and single-nuclei RNA sequencing has enabled the characterization of a broad range of cells from a heterogenous tissue. We applied this technology to garner an understanding of the channel catfish spleen and develop an atlas that can be used for additional investigation. The spleen is considered a major lymphoid organ that filters pathogens and antigens from the blood and maintains an environment for the immune system to operate. Therefore, this atlas further provides an insight into the gene expression of erythrocytes and immune cells in this evolutionary and commercially relevant teleost species.

## Supporting information

S1 Fig Sample contribution to integrated cluster analysis. (a) tSNE map (b) Bar plot demonstrating individual sample contribution (SP1, SP2, and SP3) to the integrated dataset.
(TIF)

S2 Fig Heat map of top differentially expressed genes for clusters generated from the integrated dataset. 0 = Erythroid, 1 = Erythroid, 2 = Erythroid, 3 = Erythroid, 4 = HSC, 5 = B Cells, 6 = Erythroid, 7 = Erythroid, 8 = T/NK Cells, 9 = B Cells, 10 = HSC, 11 = Myeloid, 12 = B Cells, 13 = B Cells, 14 = Myeloid, 15 = Endothelial Cells, 16 = Myeloid.
(TIF)

S3 Fig Subcluster analysis of immune cells only (no erythroid cells) using the integrated dataset. (a) tSNE plot (b) Average expression (z-score) of cell type markers (x-axis) for each cluster (y-axis). The size of the dot represents the percentage of cells per cluster contributing to expression. The dotplot of cell markers identified these clusters as B cells, hematopoeitic stem cells (HSC), T and natural killer cells (T/NK cells), myeloid cells and endothelial cells.
(TIF)

S4 Fig Heat map of top differentially expressed genes from clusters of immune cells using the integrated dataset. 0 = B Cells, 1 = HSC, 2 = HSC, 3 = B Cells, 4 = HSC, 5 = T/NK Cells, 6 = B Cells, 7 = B Cells, 8 = Myeloid, 9 = B Cells, 10 = B Cells, 11 = T/NK Cells, 12 = Myeloid, 13 = Myeloid, 14 = Endothelial Cells, 15 = HSC.
(TIF)

S5 Fig Heat map of top differentially expressed genes from hematopoetic stem cell clusters using the integrated dataset. (TIF)

S6 Fig Heat map of top differentially expressed genes from B cell clusters using the integrated dataset. (TIF)

S7 Fig Feature maps of B cell gene expression on tSNE plots (a-x). (TIF)

S8 Fig Heat map of top differentially expressed genes from T/NK cell clusters using the integrated dataset. (TIF)

S9 Fig Feature maps of T/NK cell gene expression on tSNE plots (a-x). (TIF)

S10 Fig Heat map of top differentially expressed genes from myeloid cell clusters using the integrated dataset. (TIF)

S1 Table Top 10 differentially expressed genes for cluster generated from individual scRNAseq of spleen channel catfish samples SP1, SP2 and SP3. (XLSX)

S2 Table Top 10 differentially expressed genes for 17 clusters generated from scRNAseq of spleen from channel catfish samples (n = 3). (XLSX)

S3 Table Top 10 differentially expressed genes for sub-clusters of immune cells generated from the integrated scRNAseq of spleen from channel catfish samples (n = 3). (XLSX)

S4 Table Top 10 differentially expressed genes for sub-clusters of HSC cells generated from the integrated scRNAseq of spleen from channel catfish samples (n = 3). (XLSX)

S5 Table Top 10 differentially expressed genes for sub-clusters of B cells generated from the integrated scRNAseq of spleen from channel catfish samples (n = 3). (XLSX)

S6 Table Top 10 differentially expressed genes for sub-clusters of T and NK cells generated from the integrated scRNAseq of spleen from channel catfish samples (n = 3). (XLSX)

S7 Table Top 10 differentially expressed genes for sub-clusters of cells in the myeloid lineage generated from the integrated scRNAseq of spleen from channel catfish samples (n = 3). (XLSX)
