# Supplementary figures and images for: Single-nuclei transcriptome analysis of channel catfish spleen provides insight into the immunome of an aquaculture-relevant species
Source: PLoS One. 2024 Sep 26;19(9):e0309397. doi: 10.1371/journal.pone.0309397 (PMC11426453; doi:10.1371/journal.pone.0309397)

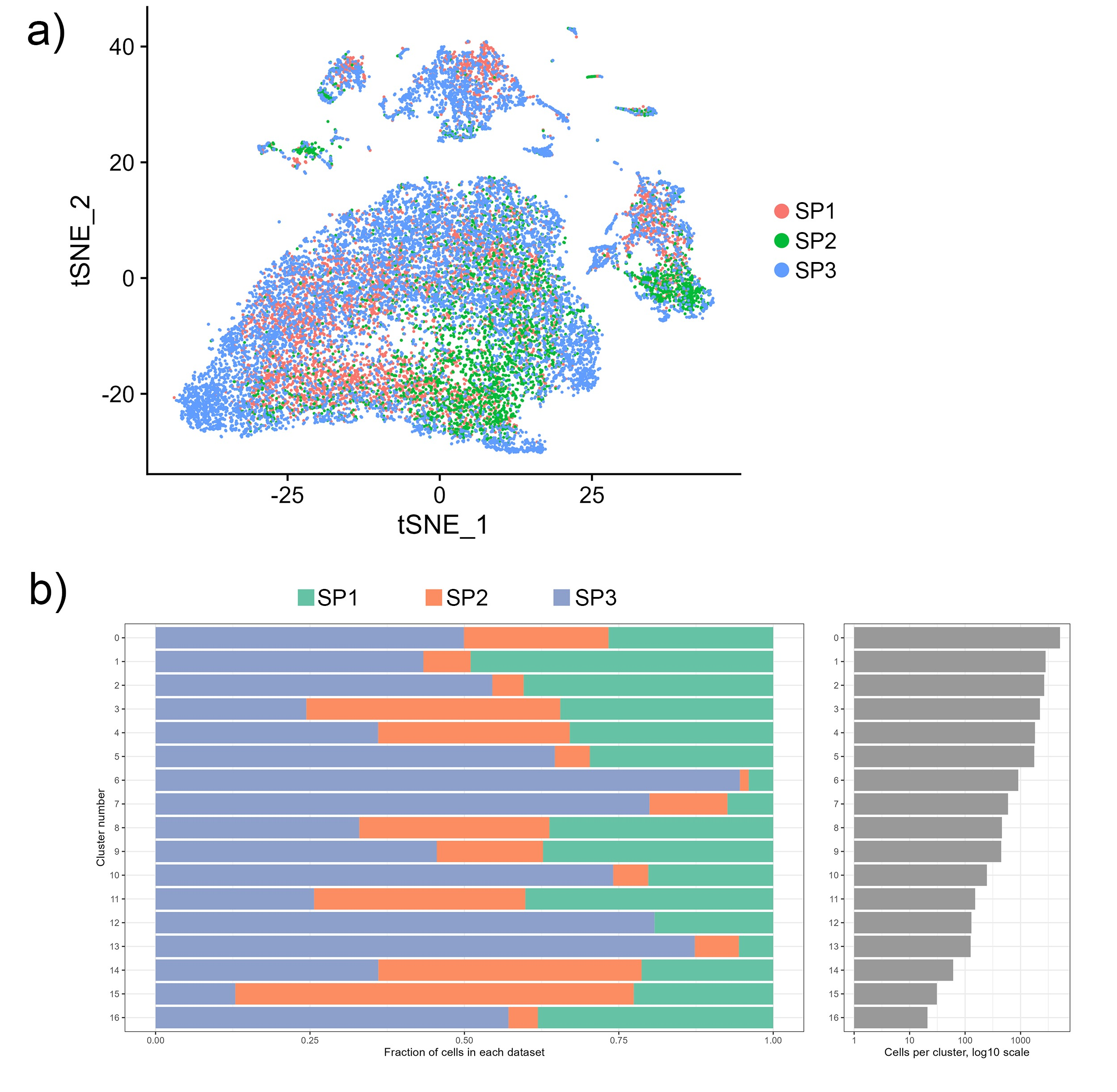

Supplement: S1 Fig — (a) tSNE map (b) Bar plot demonstrating individual sample contribution (SP1, SP2, and SP3) to the integrated dataset. (TIF) [file pone.0309397.s001.tif]

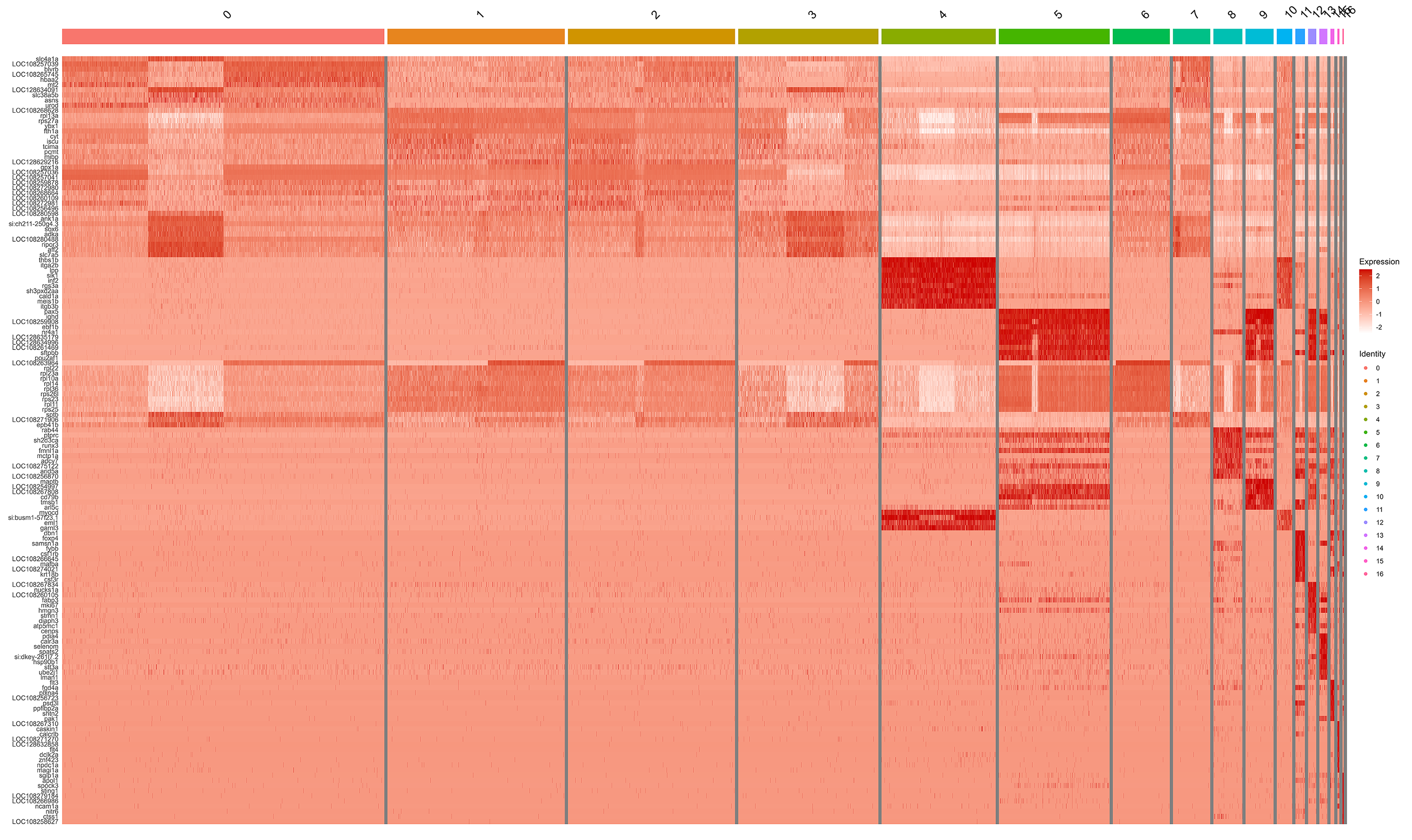

Supplement: S2 Fig — 0 = Erythroid, 1 = Erythroid, 2 = Erythroid, 3 = Erythroid, 4 = HSC, 5 = B Cells, 6 = Erythroid, 7 = Erythroid, 8 = T/NK Cells, 9 = B Cells, 10 = HSC, 11 = Myeloid, 12 = B Cells, 13 = B Cells, 14 = Myeloid, 15 = Endothelial Cells, 16 = Myeloid. (TIF) [file pone.0309397.s002.tif]

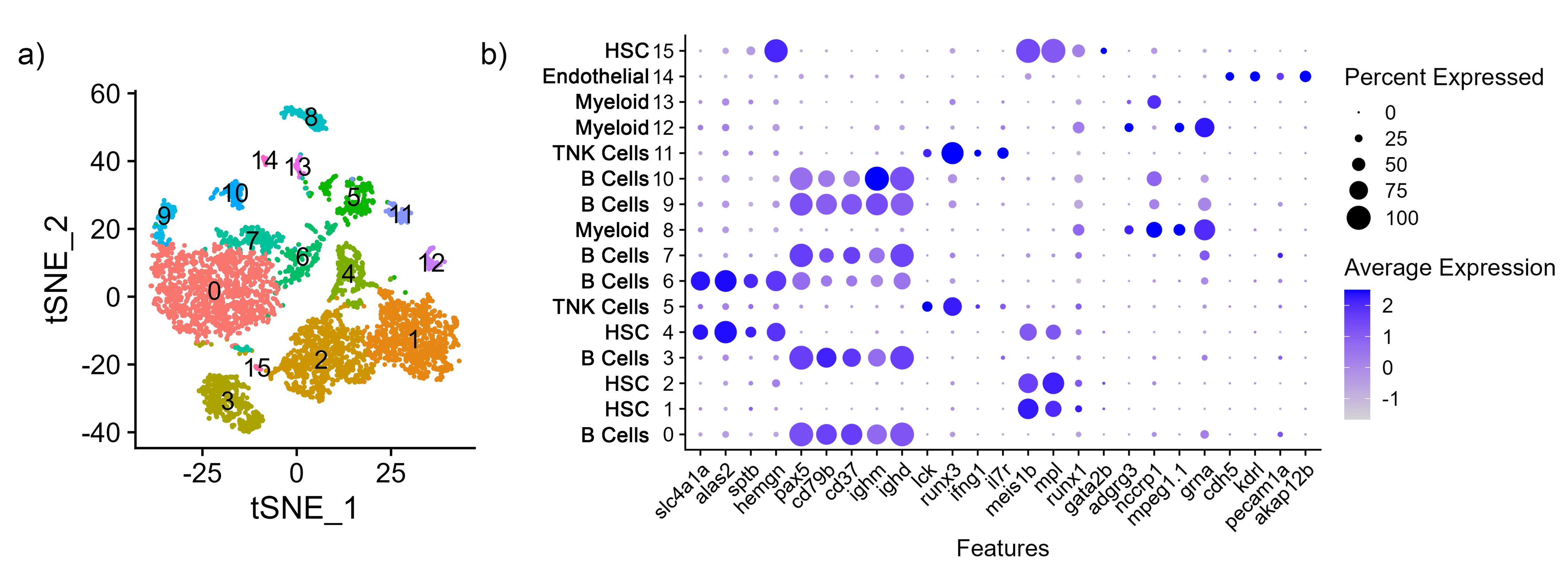

Supplement: S3 Fig — (a) tSNE plot (b) Average expression (z-score) of cell type markers (x-axis) for each cluster (y-axis). The size of the dot represents the percentage of cells per cluster contributing to expression. The dotplot of cell markers identified these clusters as B cells, hematopoeitic stem cells (HSC), T and natural killer cells (T/NK cells), myeloid cells and endothelial cells. (TIF) [file pone.0309397.s003.tif]

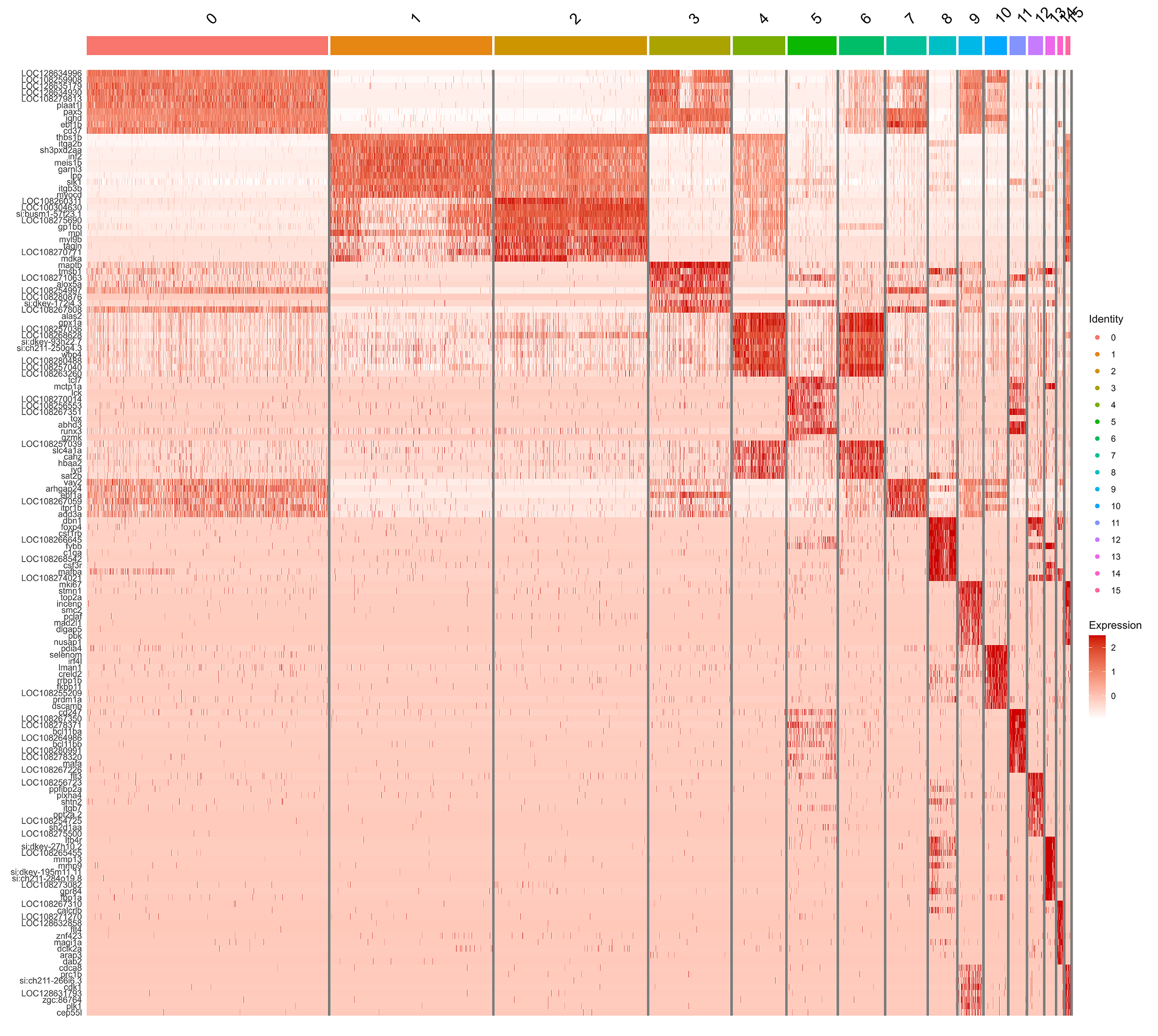

Supplement: S4 Fig — 0 = B Cells, 1 = HSC, 2 = HSC, 3 = B Cells, 4 = HSC, 5 = T/NK Cells, 6 = B Cells, 7 = B Cells, 8 = Myeloid, 9 = B Cells, 10 = B Cells, 11 = T/NK Cells, 12 = Myeloid, 13 = Myeloid, 14 = Endothelial Cells, 15 = HSC. (TIF) [file pone.0309397.s004.tif]

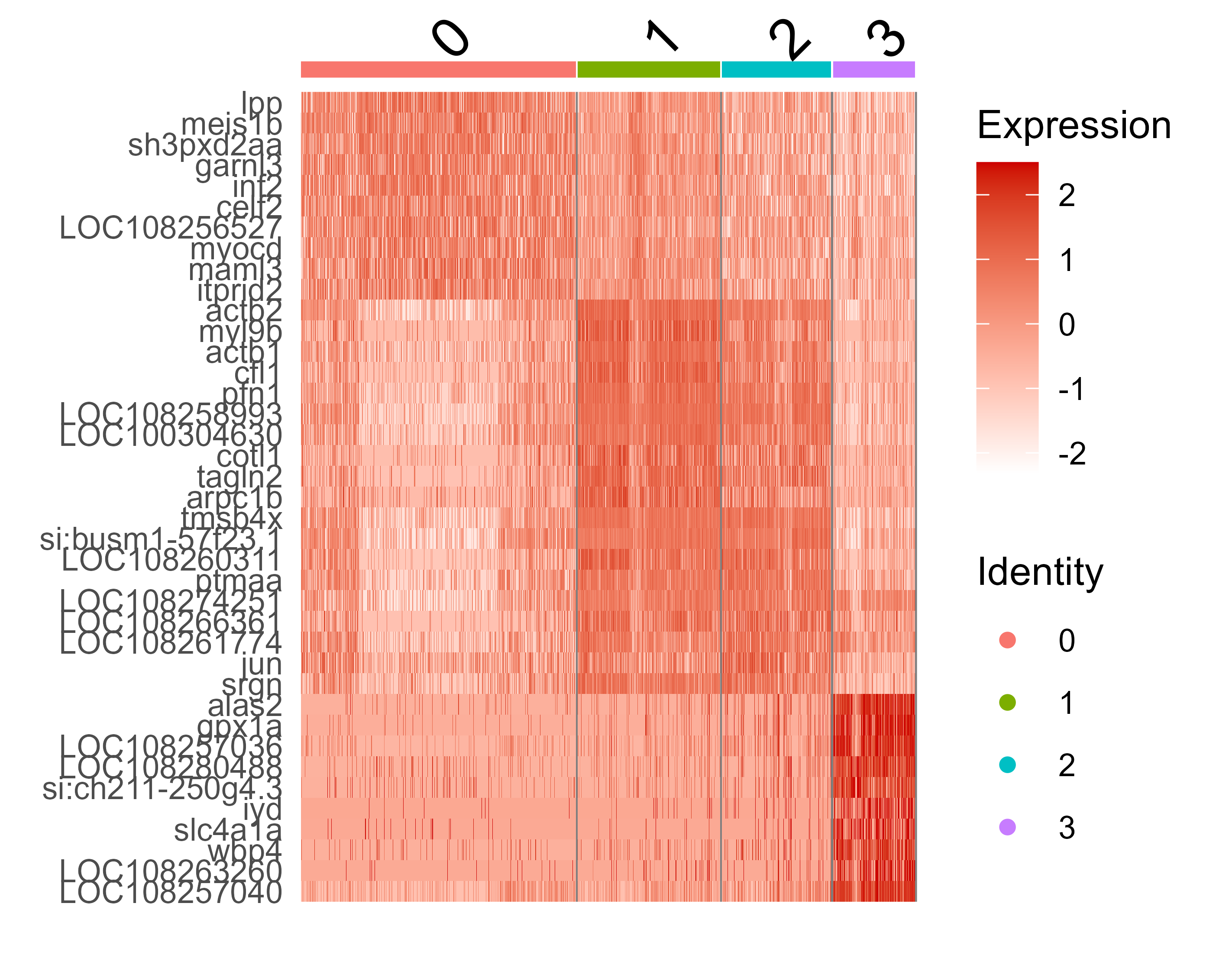

Supplement: S5 Fig — (TIF) [file pone.0309397.s005.tif]

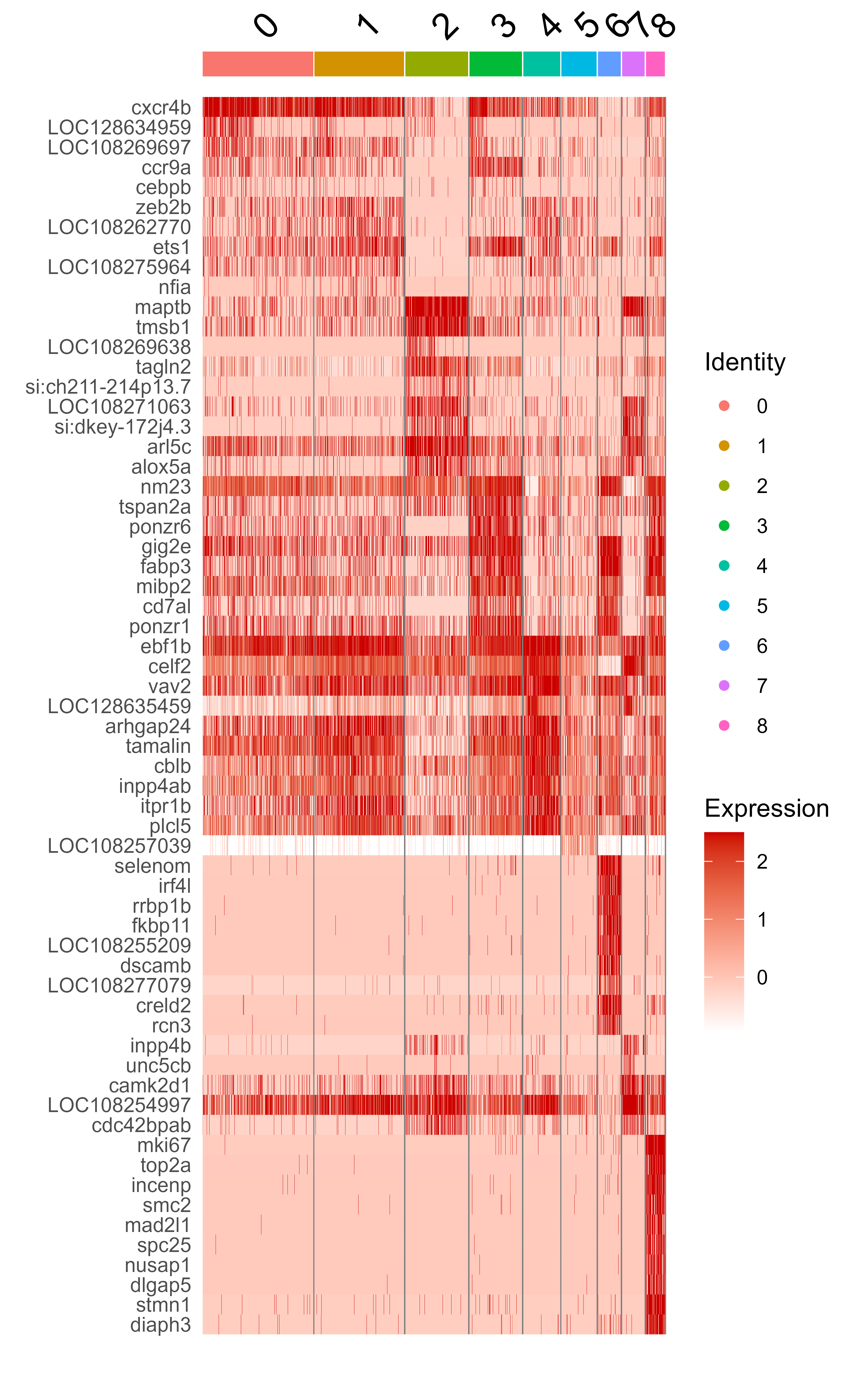

Supplement: S6 Fig — (TIF) [file pone.0309397.s006.tif]

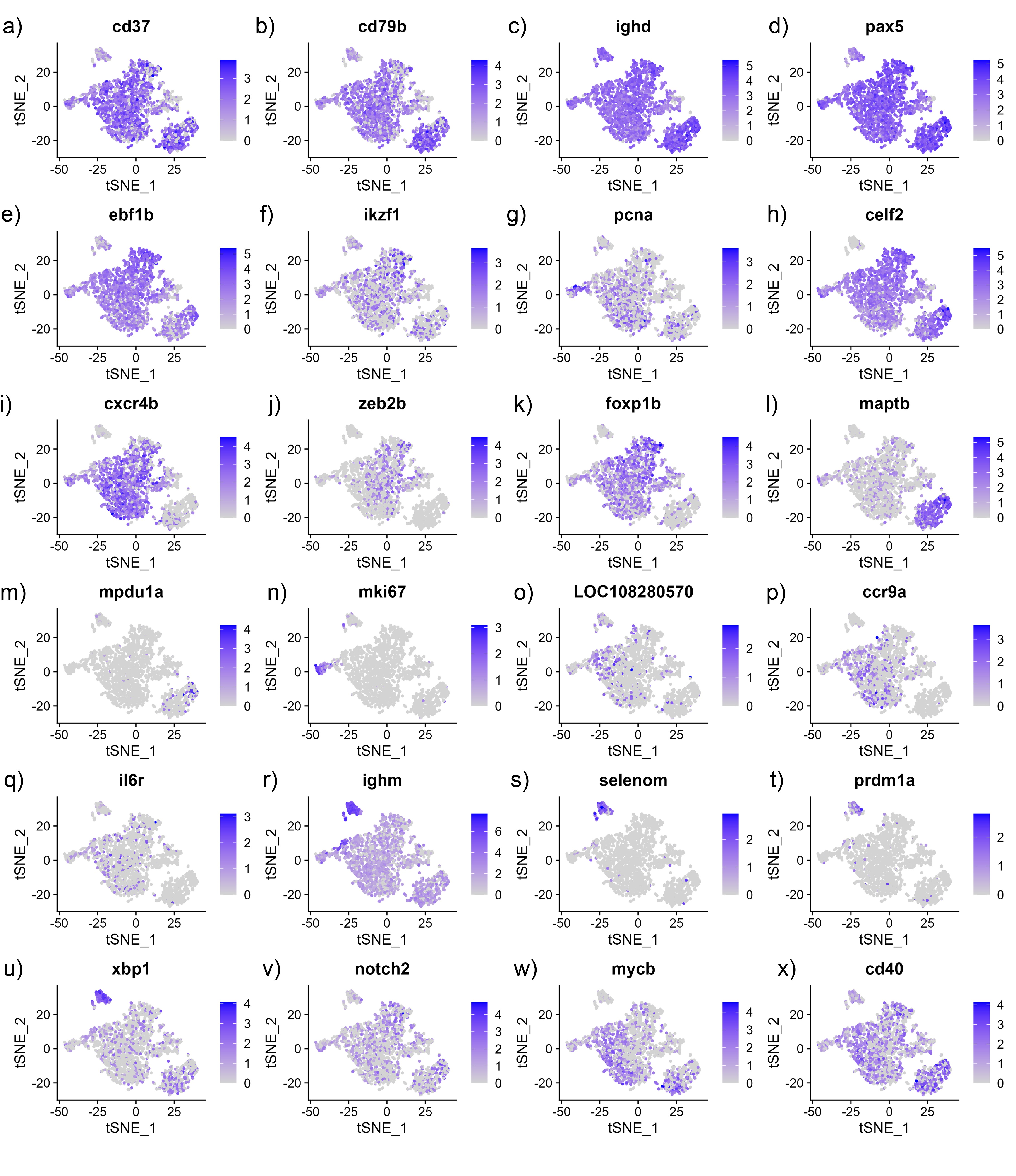

Supplement: S7 Fig — (TIF) [file pone.0309397.s007.tif]

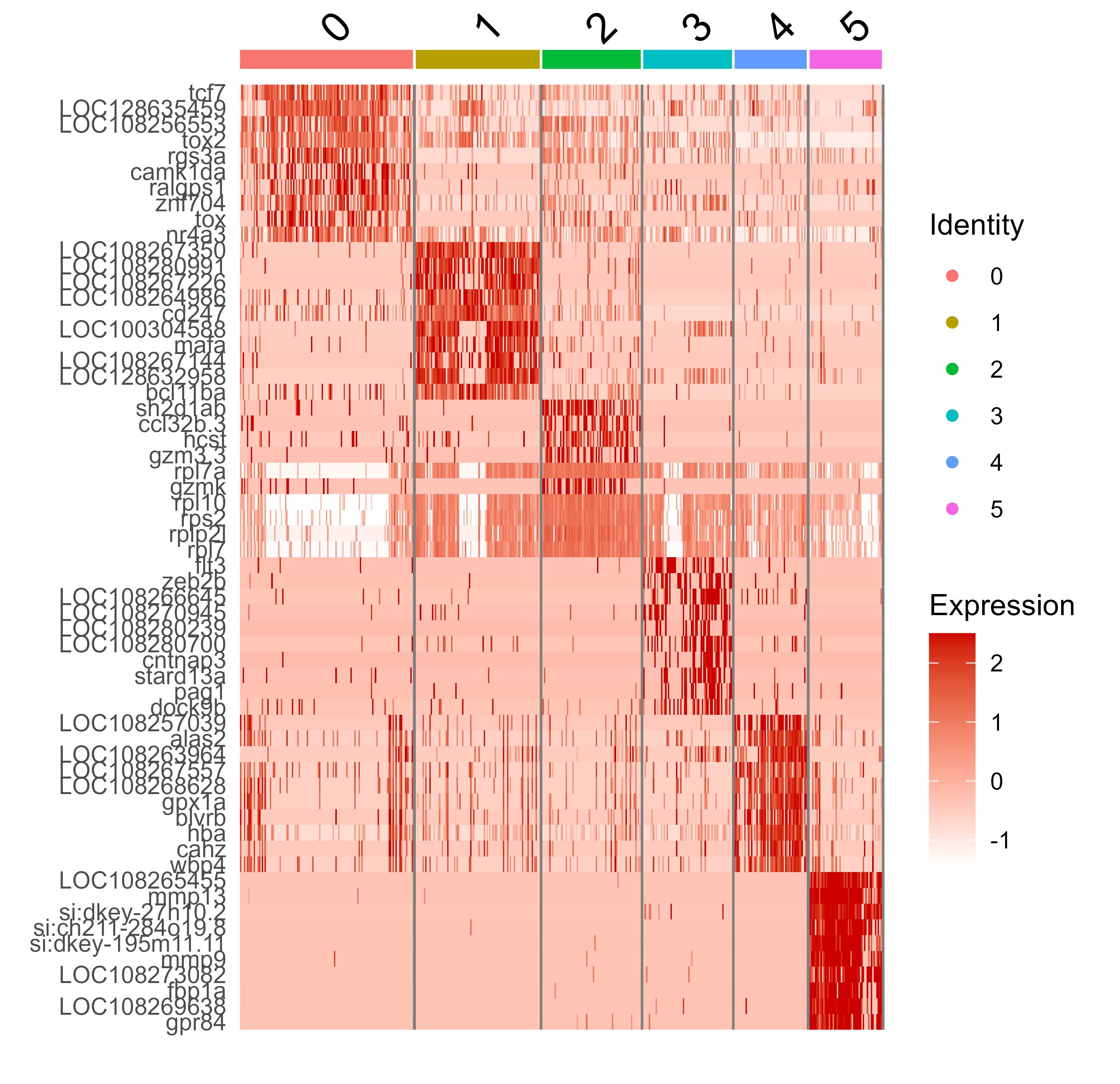

Supplement: S8 Fig — (TIF) [file pone.0309397.s008.tif]

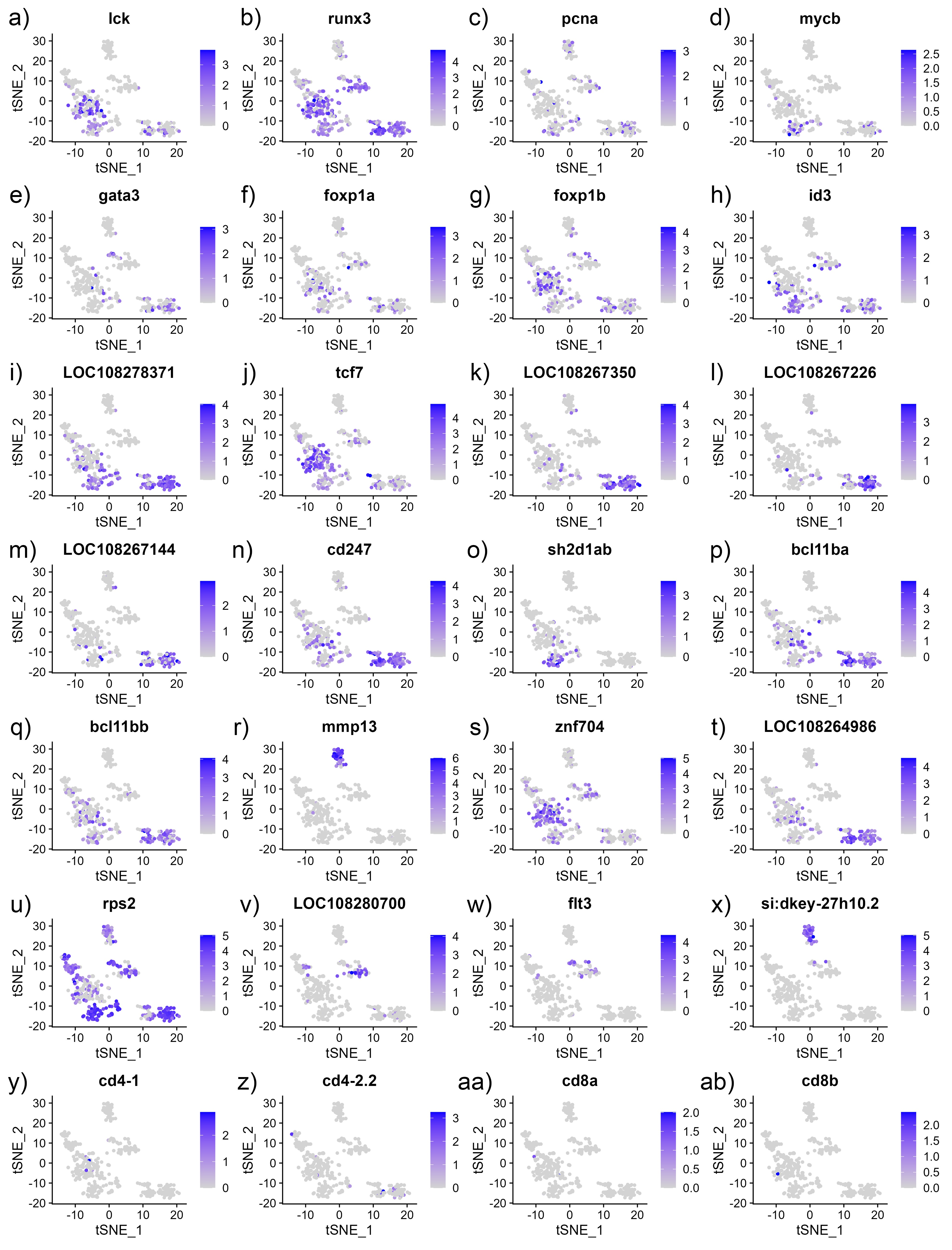

Supplement: S9 Fig — (TIF) [file pone.0309397.s009.tif]

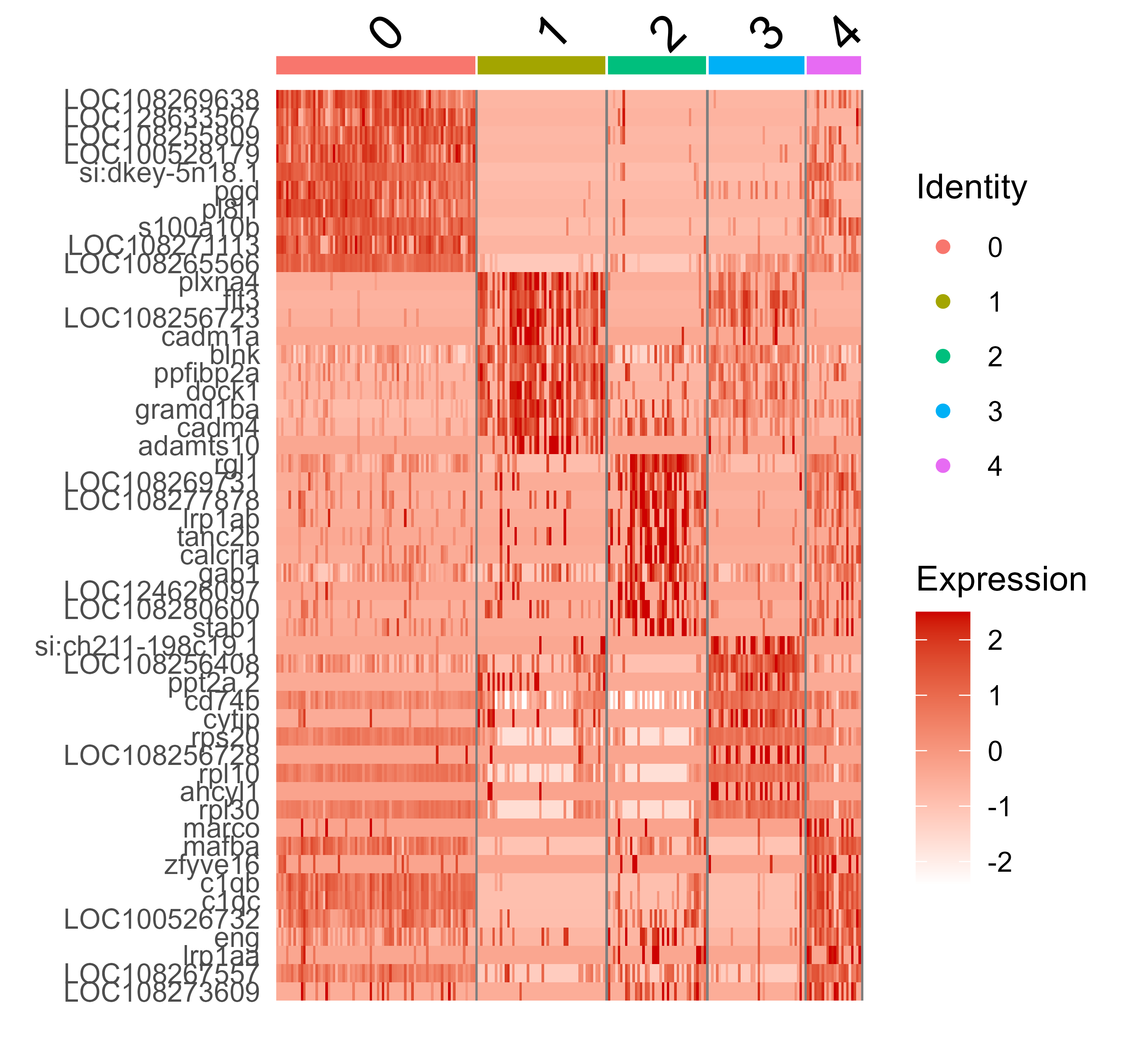

Supplement: S10 Fig — (TIF) [file pone.0309397.s010.tif]
